# Elimination of N-glycosylation by site mutation further prolongs the half-life of IFN-α/Fc fusion proteins expressed in *Pichia pastoris*

**DOI:** 10.1186/s12934-016-0601-9

**Published:** 2016-12-07

**Authors:** Hao Jia, Yugang Guo, Xiaoping Song, Changsheng Shao, Jing Wu, Jiajia Ma, Mingyang Shi, Yuhui Miao, Rui Li, Dong Wang, Zhigang Tian, Weihua Xiao

**Affiliations:** 1 The CAS Key Laboratory of Innate Immunity and Chronic Disease, Innovation Center for Cell Signaling Network, School of Life Sciences, University of Science and Technology of China, Hefei, China; 2 Hefei National Laboratory for Physical Sciences at the Microscale, Engineering Technology Research Center of Biotechnology Drugs, Anhui Province, University of Science and Technology of China, Hefei, China; 3 Anhui Engineering Research Center of Recombinant Protein Pharmaceutical Biotechnology, Institute of Advanced Technology, University of Science and Technology of China, Hefei, China; 4 Department of Pharmacy, Anhui Medical College, Hefei, China

**Keywords:** IFN-α/Fc, Fusion protein, Glycosylation, Circulation half-life, *Pichia pastoris*

## Abstract

**Background:**

Interferon (IFN)-α has been commonly used as an antiviral drug worldwide; however, its short half-life in circulation due to its low molecular weight and sensitivity to proteases impacts its efficacy and patient compliance.

**Results:**

In this study, we present an IgG1 Fc fusion strategy to improve the circulation half-life of IFN-α. Three different forms of IgG1 Fc fragments, including the wild type, aglycosylated homodimer and aglycosylated single chain, were each fused with IFN-α and designated as IFN-α/Fc-WT, IFN-α/Fc-MD, and IFN-α/Fc-SC, respectively. The recombinant proteins were expressed in *Pichia pastoris* and tested using antiviral and pharmacokinetic assays in comparison with the commercial pegylated-IFN-α (PEG-IFN-α). The in vitro study demonstrated that IFN-α/Fc-SC has the highest antiviral activity, while IFN-α/Fc-WT and IFN-α/Fc-MD exhibited antiviral activities comparable to that of PEG-IFN-α. The in vivo pharmacokinetic assay showed that both IFN-α/Fc-WT and IFN-α/Fc-MD have a longer half-life than PEG-IFN-α in SD rats, but IFN-α/Fc-SC has the shortest half-life among them. Importantly, the circulating half-life of 68.3 h for IFN-α/Fc-MD was significantly longer than those of 38.2 h for IFN-α/Fc-WT and 22.2 h for PEG-IFN-α.

**Conclusions:**

The results demonstrate that the elimination of N-glycosylation by mutation of putative N-glycosylation site further prolongs the half-life of the IFN-α/Fc fusion protein and could present an alternative strategy for extending the half-life of low-molecular-weight proteins expressed by *P. pastoris* for in vivo studies as well as for future clinical applications.

**Electronic supplementary material:**

The online version of this article (doi:10.1186/s12934-016-0601-9) contains supplementary material, which is available to authorized users.

## Background

Interferon (IFN)-α belongs to a class of cytokines that play an important role in host defenses due to their potent antiviral, immunomodulatory, and anti-proliferative activities [1]. IFN-α is commonly used to treat chronic hepatitis B and C virus (HBV and HCV) infections and certain cancers [2, 3]. The conventional IFN-α with a small molecular size of approximately 18 kDa has a short half-life of approximately 2–3 h because of its high sensitivity to serum proteolytic enzymes and rapid clearance by the kidneys [4]. Thus, patients must experience frequent injections and fluctuations in plasma drug concentration, which are associated with common adverse events such as fever, headache, and chill, especially in long-term treatment. To overcome this problem, two pegylated versions of IFN-α have been introduced with an extended half-life of 35–77 h in patients and a reduced injection frequency of once weekly [1]. Moreover, human albumin-fused IFN-α, called Albuferon, has a longer half-life of up to 120 h, which would allow for administration once every 2–4 weeks. Unfortunately, Albuferon has not yet been approved because of safety concerns at high doses [5]. Several forms of human IgG Fc-fused IFN-α fusion proteins expressed in mammalian cell lines have produced promising results in preclinical studies [2, 5, 6]. In fact, more than ten therapeutic Fc fusion proteins have been approved for clinical use by the FDA, and some of these proteins have achieved great success on the market, such as Enbrel (TNFR-Fc) [7]. Moreover, three ‘biobetters’ of Fc fusion proteins, including Eloctate (Factor VIII-Fc), were approved by the FDA in 2014 [7]. These successful cases greatly inspire our confidence in the use of Fc fusion proteins to extend the serum half-life of IFN-α.

There is a specific asparagine-linked glycosylation site (297 N) in each Fc region of heavy chains of IgG1 [8], and the absence of glycosylation at 297 N of Fc is not required for neonatal FcRn binding [9]. Thus, the glycosylation site could be mutated to eliminate N-glycosylation in Fc when expressed in the yeast *Pichia pastoris,* an expression system commonly used to control the cost of production [10]. Moreover, it is reported that monomeric Fc binds to human FcRn at a rate comparable to that of normal Fc [11], and IFN-α appears to biologically function as a monomer [12]. Thus, in this study, three different IFN-α/Fc fusion proteins, including IFN-α fused to the wild-type IgG1 Fc fragment (IFN-α/Fc-WT), IFN-α fused to a modified IgG1 Fc fragment (IFN-α/Fc-MD) and IFN-α fused to a modified single-chain IgG1 Fc fragment (IFN-α/Fc-SC), were designed and expressed. To evaluate the in vitro properties and the antiviral and anti-proliferative activities of IFN-α/Fc fusion proteins, conventional IFN-α and pegylated IFN-α (PEG-IFN-α, Pegasys) were compared in several cell lines. To evaluate the in vivo properties of the fusion proteins, a pharmacokinetic study was conducted in rats. The objective of this study was to provide one or two possible forms of IFN-α/Fc fusion proteins for further applications.

## Methods

### Strains, plasmids, antibodies and equipment

*Pichia pastoris* strain GS115, *Escherichia coli* strain DH5α, and expression vector pPIC9 were purchased from Invitrogen (Life Technologies). MDBK and Daudi cells were obtained from Wuhan Boster. The plasmid pPIC9K-Kex-IFNα2b-Fcγ1 with the coding gene of fused human IFN-α2b and the wild-type human IgG1 Fc fragment was constructed and preserved in our laboratory [13]. WISH cells, the conventional IFN-α and the anti-IFN-α monoclonal antibody were obtained from Anke Biotechnology Group. HPR-conjugated anti-mouse antibodies were purchased from Cell Signaling. The 14-L fermenter (New Brunswick BioFlo115) used in pilot-scale fermentation was obtained from Eppendorf. A flex stand system with 0.45-μm hollow fiber membrane filtration cartridges and an AKTA Avant system with MabSelect and S-200 HR columns were obtained from GE Healthcare.

### Molecular design and cloning

The fusion protein IFN-α/Fc-WT was secreted into culture medium as the active form of disulfide-linked homodimer with a single N-glycosylation modification in the IgG1 Fc region in each molecule. Overlap extension PCR was used for site-specific mutagenesis of the glycosylation site on the IgG1 Fc fragment and for the insertion of a flexible GS linker between the C-terminus of IFN-α and the N-terminus of the IgG1 Fc fragment.

The primer pairs (see Additional file 1: Table S1) α-Fw and SC-Rv, SC-Fw and M-Rv, and M-Fw and Fc-Rv were used to amplify three parts of the fused coding sequence of the IFN-α/Fc-SC monomer on the template plasmid pPIC9K-Kex-IFNα2b-Fcγ1 (pIFN-α/Fc-WT), which was used for expression of the fusion protein IFN-α/Fc-WT. The PCR procedure was as follows: 94 °C for 5 min; 32 cycles of 94 °C for 30 s, 55 °C for 40 s, and 72 °C for 60 s; and 72 °C for 10 min. The primer pair α-Fw and Fc-Rv was used to splice the three amplified parts to obtain the complete fused IFN-α2b and the IgG1 gene. The PCR procedure was as follows: 94 °C for 5 min; 32 cycles of 94 °C for 30 s, 55 °C for 40 s, and 72 °C for 120 s; and 72 °C for 10 min. This newly amplified gene encoding a single-chain IFN-α2b and the IgG1 Fc fragment-fused protein contained a mutated glycosylation site on the IgG1 Fc fragment (297 N to Q) with the insertion of a GS linker between IFN-α2b and the IgG1 Fc fragment. The amplified gene was digested with the restriction enzymes *Bam*H I and *Eco*R I and was inserted into the same digested pPIC9 vector. This recombinant expression vector is denoted as pIFN-α/Fc-SC.

The primer pairs α-Fw and MD-Rv and MD-Fw and Fc-Rv were used to amplify two parts of the fused coding sequences of the IFN-α/Fc-MD homodimer on the template plasmid pIFN-α/Fc-SC. The PCR procedure was as follows: 94 °C for 5 min; 32 cycles of 94 °C for 30 s, 55 °C for 40 s, and 72 °C for 60 s; and 72 °C for 10 min. The primer pair α-Fw and Fc-Rv was used to splice the two amplified parts to obtain the complete fused IFN-α2b and the IgG1 gene. The PCR procedure was as follows: 94 °C for 5 min; 32 cycles of 94 °C for 30 s, 55 °C for 40 s, and 72 °C for 120 s; and 72 °C for 10 min. This newly amplified gene encoding a homo-dimerized IFN-α2b and the IgG1 Fc fragment-fused protein also contained a mutated glycosylation site on the IgG1 Fc fragment with the insertion of a protein linker between IFN-α2b and the IgG1 Fc fragment. This newly amplified gene was digested with the restriction enzymes *Bam*H I and *Eco*R I and was inserted into the same digested pPIC9 vector. This recombinant expression vector was denoted as pIFN-α/Fc-MD.

### Screening for expression

The recombinant expression vectors pIFN-α/Fc-WT, pIFN-α/Fc-MD and pIFN-α/Fc-SC were linearized by the restriction enzyme *Sal *I. The linearized vectors were transformed into *P. pastoris* strain GS115. The positive transformants were screened by MD plates and examined by Western blot using anti-human IgG-HRP conjugates according to a previous report [14].

### Fermentation and purification

The fed-batch fermentation process was performed in a 14 L fermentor (NBS BioFlo 115) with 6 L BMGY media containing 4% glycerol according to previous reports with necessary modifications [15]. Specifically, the culture temperature was maintained at 25 °C, and the pH was controlled at 6.0 with ammonium hydroxide. Moreover, after induction by 100% methanol supplemented with 12 mL/L of PTM1 solution, the DO was set to about 30% air saturation and fermentation was allowed to proceed for less than 18 h to reduce degradation of the targeted products. At the end of fermentation, the pH of the fermentation broth was adjusted to 8.0 by 5 M NaOH and then centrifuged for 20 min at 10,000* g*. After centrifugation, the supernatant was filtered through 0.45-μm hollow fiber membranes using the FlexStand benchtop system. A HiTrap MabSelect affinity chromatography column was used to capture the recombinant IFN-α/Fc-fused proteins from the clarified supernatants. The captured proteins were further purified with HiPrep Sephacryl S-200 HR size-exclusion chromatography and stored at −80 °C.

### Characterization of the fusion proteins

SDS-PAGE under reduced and non-reduced conditions was performed to analyze the purified proteins. Approximately 10 μg of each IFN-α/Fc fusion protein was loaded and separated by 10% SDS-PAGE; the gel was stained with Coomassie brilliant blue. Western blotting was also performed using the anti-IFN-α monoclonal antibody or anti-human IgG-HRP conjugates. The protein samples were separated by 10% SDS-PAGE under reduced conditions and then transferred to a PVDF membrane for 30 min at 18 V. The membrane was blocked with 5% skim milk for 1 h and then incubated with the indicated antibodies. The membrane was washed three times with TBST and then incubated with anti-mouse HRP-conjugated secondary antibodies. The protein was detected using a chemiluminescence detection kit after the membrane was washed three times with TBST. A Periodic acid-Schiff (PAS) staining kit (Catalog No. DG0005, Beijing Leagene Biotechnology) was used according to the manufacturer’s instructions to characterize the glycosylated modification of the fusion proteins. The recombinant proteins were also analyzed by liquid chromatography–mass spectrometry (LC–MS), which was performed as previously described [16].

### Antiviral activity assay

WISH or MDBK cells were diluted with DMEM containing 10% fetal bovine serum (FBS) to 2.5 × 10^5^–3.5 × 10^5^ cells/mL. Each well of a 96-well plate was seeded with 100 μL of these diluted cells. Approximately 6 h later, the medium was replaced with DMEM containing 7% FBS and serially diluted samples. After 24 h, the medium was replaced with DMEM containing 3% FBS and 100 TCID_50_ VSV. Another 24 h later, the cell viability was measured using an MTT assay. The activities of different samples were calculated using Origin 8 software. The experiments were run in triplicate.

### Anti-proliferation assay

Daudi cells were diluted with DMEM containing 10% FBS to 5 × 10^5^ cells/mL. Each well of a 96-well plate was seeded with 50 μL of the diluted cells. Then, 50 μL of DMEM containing 10% FBS was added, and the samples were serially diluted. After 72 h, the cell viability was measured using the MTT assay. The EC_50_ values of different samples were calculated using Origin 8 software. The experiments were run in triplicate.

### 2′, 5′-oligoadenylate synthetase (OAS) mRNA assay

Human PBMCs were isolated from the peripheral blood of healthy adult volunteers using Ficoll density gradient centrifugation and diluted with RPMI 1640 medium containing 10% FBS to 1.5 × 10^5^–2.0 × 10^5^ cells/mL. Each well of a 6-microwell plate was seeded with 2 mL of the diluted cells. After 12 h of incubation, the indicated samples were added to each well at a final concentration of 10 ng/mL. After 20 h of incubation, the total RNA of the cultured cells was isolated with TRIzol extraction. Real-time quantitative RT-PCR was used to detect the expression of the targeted genes according to a previous report [1].

### Pharmacokinetics study

Fifteen Sprague–Dawley (SD) rats with weights ranging from 180 to 220 g were randomly and equally divided into five groups. A 20% ethyl carbamate solution at a dose of 5 mL/kg was used for animal anesthesia. After anesthesia, the indicated samples were injected intravenously into corresponding animal groups with a single 30 μg/kg dose. Blood samples of the treated group were collected prior to dosing and at the following times after dosing: 0.2, 8, 24, 48, 72, 96, 120 and 144 h. Heparin sodium was used for anticoagulation, and then the blood samples were centrifuged for plasma harvest. The interferon levels of the blood samples were analyzed using cytometric bead array (CBA) according to the manufacturer’s instructions. The pharmacokinetic parameters were calculated using the software PKsolver with a non-compartmental model [17].

### Statistical analysis

Difference between groups was analyzed by applying the one-way analysis of variance (ANOVA) with Tukey’s test (P < 0.05 as significant, P < 0.01 as highly significant, P < 0.001 as very highly significant).

## Results

### Molecular design of IFN-α/Fc fusion proteins

**Fig. 1:**
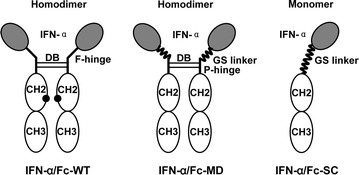
Schematic diagram for IFN-α/Fc fusion proteins. The dimer is composed of two molecules of IFN-α joined to dimeric Fc, and the monomer has a single molecule of IFN-α linked to monomeric Fc. *Black circles* N-glycosylation site in the wild-type IgG1 Fc region; *DB* disulfide bridges between dimeric Fc; *F-hinge* full hinge region of IgG1; *P-hinge* partial hinge with the amino acid sequence of HTCPPCP

In this study, all three genes were under the control of the classical alcohol oxidase 1 (AOX1) promoter, and their expression was induced by methanol in *P. pastoris*. Schematic diagrams of the fusion proteins are shown in Fig. 1. Compared to IFN-α/Fc-WT, the glycosylation site on the IgG1 Fc fragment was mutated (297 N to Q), and a flexible GS linker replaced the partial hinge (eight amino acids of the N-terminus of the hinge) of IgG1 Fc in IFN-α/Fc-MD. IFN-α/Fc-SC became a monomeric fusion protein via replacement of the full hinge region with the GS linker.

### Expression of IFN-α/Fc fusion proteins

**Fig. 2:**
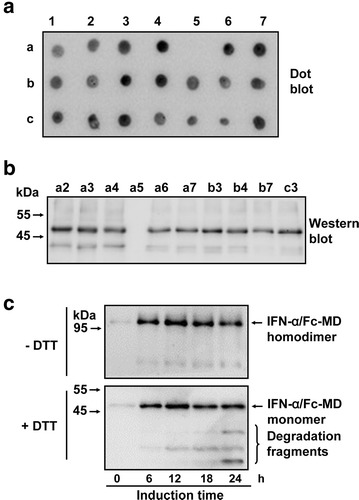
Expression of the IFN-α/Fc-MD fusion protein. The transformed colonies of IFN-α/Fc-MD were screened by dot blot (**a**); and further confirmed by Western blot (**b**). a1–c8: the colony number. **c** The time period for the expression of IFN-α/Fc-MD during induction in fermentation was analyzed by Western blot under reduced and non-reduced conditions

The three recombinant expression vectors IFN-α/Fc-WT, IFN-α/Fc-MD and IFN-α/Fc-SC were each transformed into *P. pastoris* strain GS115. The process and results of transforming, screening and expressing were similar for each IFN-α/Fc fusion protein. For example, the positive transformants were first screened by dot blot (Fig. 2a), and then, the selected high-expression clones were further confirmed by Western blot (Fig. 2b). One of these high-expression strains was used in pilot-scale fermentation. As shown in Fig. 2c, the IFN-α/Fc-MD fusion protein began to accumulate after induction by methanol in fermentation. This induction was allowed to proceed for less than 18 h to prevent degradation of the targeted products.

### Purification and characterization of IFN-α/Fc fusion proteins

**Fig. 3:**
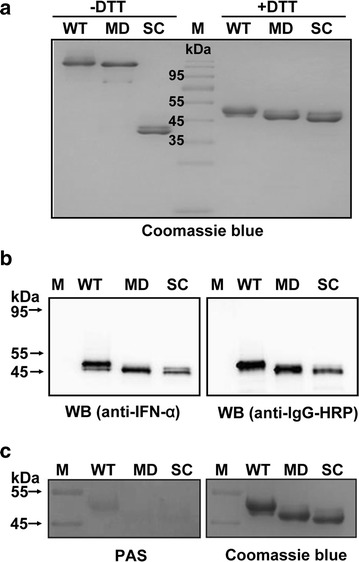
Characterization of purified IFN-α/Fc fusion proteins. **a** Comparison of purified IFN-α/Fc fusion proteins using SDS-PAGE under reduced and non-reduced conditions. **b** Western blot analysis of the purified IFN-α/Fc fusion proteins. The indicated samples were separated via reduced SDS-PAGE and identified through Western blot analysis with goat anti-human IgG-HRP conjugate or mouse anti-human IFN-α monoclonal antibodies, followed by rabbit anti-mouse IgG-HRP conjugate. **c** PAS staining of purified IFN-α/Fc fusion proteins via SDS-PAGE under reduced conditions. WT: IFN-α/Fc-WT, MD: IFN-α/Fc-MD, SC: IFN-α/Fc-SC, M: prestained protein marker

After fermentation, the recombinant proteins were captured using HiTrap MabSelect affinity chromatography, which is specifically used for the purification of Fc fusion protein or antibodies. HiPrep Sephacryl S-200 size exclusion chromatography was used for polishing purification. The purity of the recombinant proteins was greater than 90% when examined by SDS-PAGE, as shown in Fig. 3a. In addition, the predicted molecular weight (MW) of IFN-α/Fc-WT, IFN-α/Fc-MD and IFN-α/Fc-SC is 90.8, 90.0 and 44.4 kDa, respectively. The apparent MW of IFN-α/Fc-SC under non-reduced conditions was approximately half that of IFN-α/Fc-WT and IFN-α/Fc-MD, but under reduced conditions, the apparent MW was close to that of IFN-α/Fc-WT and IFN-α/Fc-MD, indicating that IFN-α/Fc-SC is a monomeric fusion protein. Western blot was performed to demonstrate that both IFN-α and the antibody Fc fragment were present in the fusion proteins, as shown in Fig. 3b. The fusion proteins were also confirmed by subsequent LC-MS peptide mapping (data not shown). PAS staining and Coomassie brilliant blue staining of the same SDS-PAGE gel collectively showed that site-specific mutagenesis of the N-glycosylation site in the Fc region can dramatically reduce the glycosylation level of the fusion proteins (Fig. 3c).

### Anti-viral activity of IFN-α/Fc fusion proteins

**Fig. 4:**
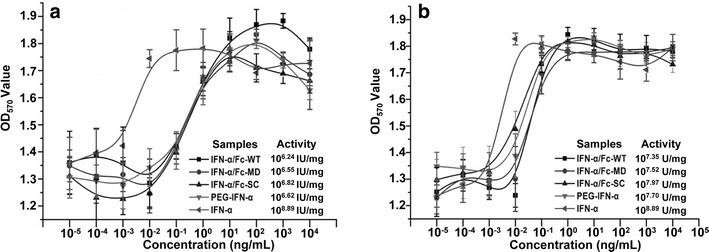
Antiviral activity of IFN-α/Fc fusion proteins. The protective effects of IFN-α/Fc fusion proteins and two controls (recombinant human IFN-α and pegylated interferon PEG-IFN-α) at the indicated concentrations were evaluated in antiviral assays using human WISH (**a**) and bovine MDBK (**b**) challenged with the VSV virus. All data are presented here as mean ± standard deviation of the OD values

Two common in vitro test systems were used to evaluate the anti-viral activity of the IFN-α/Fc fusion proteins. In the WISH-VSV system, conventional IFN-α exhibited the significantly higher anti-viral activity, comparing with the three IFN-α/Fc-fused proteins and PEG-IFN-α (*P* < 0.001), and three IFN-α/Fc-fused proteins showed similar anti-viral activity compared to PEG-IFN-α (Fig. 4a). At 10^6.82^ IU/mg, the anti-viral activity of IFN-α/Fc-SC was slightly higher than that of IFN-α/Fc-WT, IFN-α/Fc-MD and PEG-IFN-α, but this difference did not reach statistical significance (*P* > 0.05). In the MDBK-VSV system (Fig. 4b), similarly, conventional IFN-α still exhibited the significantly higher anti-viral activity comparing with the three IFN-α/Fc-fused proteins and PEG-IFN-α (*P* < 0.001) and IFN-α/Fc-SC showed the higher anti-viral activity than IFN-α/Fc-WT and IFN-α/Fc-MD but the difference was still not significant (*P* > 0.05). Human PBMCs were treated with 10 ng/mL of the indicated samples. The total RNA was extracted, and real-time quantitative RT-PCR was used to evaluate the transcription levels of OAS1 and STAT1 (Table 1). With respect to the OAS1 transcription level, three IFN-α/Fc fusion proteins in this study showed similar activities, which were less than that of PEG-IFN-α. There was no dramatic difference in the transcription of STAT1 among the IFN-α/Fc fusion proteins and PEG-IFN-α.

**Table 1:** Induction of mRNA expression by IFN-α/Fc fusion proteins in PBMCs

Samples	OAS1	STAT1
IFN-α	19.59	1.67
IFN-α/Fc-WT	10.47	1.34
IFN-α/Fc-MD	10.60	1.41
IFN-α/Fc-SC	10.75	1.23
PEG-IFN-α	11.73	1.26
PBS	1.00	1.00

PBMCs were treated with 10 ng/mL of indicated samples. RNA was extracted and the level of induction was measured relative to untreated cells (PBS) by quantitative PCR

*OAS1* 2, 5-oligoadenylate synthetase 1, *STAT1* signal transducer and activator of transcription 1

### Anti-proliferation activity of IFN-α/Fc fusion proteins

**Fig. 5:**
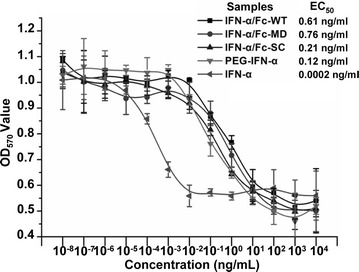
Anti-proliferative activity of IFN-α/Fc fusion proteins. Daudi cells were incubated for 72 h in the presence of increasing concentrations of the indicated samples with triple parallel wells in 96-well plates. Cell proliferation was assessed using the MTT method. All data are presented here as mean ± standard deviation of the OD values

Daudi cells were used to evaluate the anti-proliferation activity of the IFN-α/Fc fusion proteins. The anti-proliferation activities of the three IFN-α/Fc-fused proteins in this study were comparable to that of PEG-IFN-α (Fig. 5). Similar to the anti-viral activity, the anti-proliferative activity of IFN-α/Fc-SC was also slightly higher than those of IFN-α/Fc-WT and IFN-α/Fc-MD, but the difference did not reach statistical significance (*P* > 0.05).

### Pharmacokinetics study

**Fig. 6:**
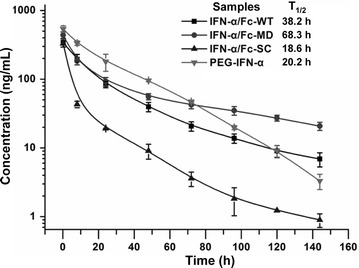
Pharmacokinetics of IFN-α/Fc fusion proteins. SD rats (n = 3 per group) were intravenously administered a single dose of 30 µg/kg of the indicated samples via the vena caudalis. Blood samples were drawn prior to treatment and at 0.2, 8, 24, 48, 72, 96, 120 and 144 h after the treatment. The serum human IFN-α level was quantified using the CBA Human IFN-α Flex Set. All data are presented here as mean ± standard deviation of the concentrations

The plasma concentration data were analyzed using a non-compartmental model, and the results are shown in Fig. 6. The half-life of IFN-α/Fc-MD was 68.3 h, much longer than that of the other samples, including PEG-IFN-α, whose half-life was 20.2 h (*P* < 0.001). The half-life of IFN-α/Fc-MD was approximately twice as long as that of IFN-α/Fc-WT. This result shows that eliminating N-glycosylation by site mutation in the IgG1 Fc region can significantly enhance the half-life of IFN-α/Fc fusion proteins. The half-life of IFN-α/Fc-SC was 18.6 h, shorter than that of the other two fusion proteins and PEG-IFN-α but still much longer than that of conventional IFN-α, whose half-life was too short to be detected at 8 h post-administration.

## Discussion

Although the two forms of pegylated IFN-α produced by pharmaceutical companies are listed as first-line antiviral drugs for chronic hepatitis B treatment by AASLD and EASL [18], for most patients with chronic hepatitis B, especially for those in China, Pegasys or PegIntron are too expensive. Consequently, patients must choose conventional IFN-α with affordable prices. Thus, it is urgent to develop an alternative IFN-α with an excellent pharmacokinetic performance and at a low cost for developing countries. In this study, three forms of active IFN-α/Fc fusion proteins were designed and successfully expressed in the *P. pastoris* expression system, which provides industrial potential for producing soluble, secreted and functional recombinant proteins with post-translational modifications at a low cost [19]. It is noted that the fermentation process needs further optimization to improve expression levels. In this study, fermentation was only performed in routine condition to collect enough proteins for this study, It is worth enough to making much effort to optimize the fermentation conditions, including optimizations of culture medium, pH, temperature, strategies of methanol feeding and so on [10, 19].

The three forms of IFN-α/Fc fusion proteins and two controls were tested for anti-viral and anti-cell proliferation activity using different in vitro test systems. Inhibiting the cytopathic effect of VSV infection in human (WISH) and bovine (MDBK) cell lines presents two classical methods for studying the anti-viral activity of IFNs [1, 20]. As expected, conventional IFN-α had the highest anti-viral activity of approximately 10^8.9^ IU/mg in both systems in our study, which is consistent with previous publications in which test systems were varied within a reasonable range [1, 5, 6]. All three IFN-α/Fc fusion proteins exhibited activities comparable to that of PEG-IFN-α. The activity of IFN-α/Fc-SC was slightly higher than those of IFN-α/Fc-WT and IFN-α/Fc-MD due to apparent steric hindrance between the IFN-α moiety and the Fc fragment [6]. IFN-α/Fc-SC may have more flexibility to facilitate binding to the corresponding receptors of IFN-α. It is noted that the IFN-α/Fc-SC in this study is a single-chain form of the IFN-α/Fc fusion protein, which is different from the previous monomeric IFN-α/Fc composed of one IFN-α molecule and the dimeric Fc domain of human IgG1 in another study [6]. Compare to the monomeric IFN-α/Fc in previous study [6], the purification process of IFN-α/Fc-SC could be much simpler because IFN-α/Fc-SC is a single molecule that can not form unwanted dimmers as found in the monomeric IFN-α/Fc [6]. Obviously, the production cost of IFN-α/Fc-SC in this study is much lower than that of the monomeric IFN-α/Fc with a heterodimeric structure. Moreover, the antiviral activity could be further improved by optimizing the linker length between IFN-α and Fc [2, 6]. OAS1 plays an important role in IFN-α-dependent anti-viral immunity [1]. The ex vivo transcription level of the OAS1 gene after interferon stimulation was examined. Consistent with the in vitro WISH-VSV and MDBK-VSV study, all IFN-α/Fc fusion proteins exhibited activities comparable to that of PEG-IFN-α.

IFN-α also functions in antitumor activity in mouse and human malignancies and has been approved as an antineoplastic therapeutic drug in clinical practice for more than 30 years [3]. The Daudi cell is a human B lymphoblast cell line that is highly sensitive to the anti-proliferative effects of IFN-α and is commonly used as an in vitro test system to evaluate the anti-proliferation activity of all types of recombinant IFN-α [1]. Similar to the anti-viral results presented above, there were comparable activities among the three IFN-α/Fc fusion proteins and PEG-IFN-α.

The pharmacokinetic study in SD rats showed that the three forms of IFN-α/Fc fusion proteins had a significantly increased half-life compared to that of conventional IFN-α, whose half-life was only approximately 0.5 h in rats [21] and rapidly decreased to undetectable levels by 8 h post-administration in this study. The half-lives of IFN-α/Fc-WT and IFN-α/Fc-MD were 38.2 and 68.3 h, respectively. These proteins showed a superior potency compared to PEG-IFN-α, with a half-life of 20.2 h in our study, consistent with other studies [21, 22]. Thus, both IFN-α/Fc-WT and IFN-α/Fc-MD possess advantages over commercial PEG-IFN-α regarding half-life elongation. Moreover, the half-life of IFN-α/Fc-MD was much longer than that of IFN-α/Fc-WT.

The N-glycosylation of recombinant proteins has been thought to have a significant impact on the pharmacokinetics of glycosylated therapeutics [23]. The post-translational glycosylation modification is usually high mannose type (mostly Man9–Man12) in wild-type *P. pastoris* [19, 24]. The IFN-α/Fc-WT was likely processed by N-glycosylated modification when expressed in *P. pastoris* (Fig. 3c). It has also been reported that the serum clearance of glycoproteins through the mannose receptor is one of the major pathways for selective glycoprotein clearance from circulation [23]. It is also reported that glycosylated mAbs with terminal high mannose glycans exhibited fast clearance from the blood [25]. High mannose-type glycosylation may reduce the half-life of IFN-α/Fc-WT expressed in *P. pastoris* through a mannose receptor on most immune cells, such as macrophages, endothelial cells and immature DCs, resulting in protease degradation instead of FcRn receptor degradation in those macrophages that recycle proteins [26, 27]. To confirm the above hypothesis, mannose receptor knockout mice could be used to further investigate the pharmacokinetics of IFN-α/Fc-WT and IFN-α/Fc-MD in the future [28, 29].

## Conclusions

In this study, elimination of N-glycosylation of IFN-α/Fc-MD by site mutation confers a much longer half-life compared to IFN-α/Fc-WT and presents another alternative strategy for enhancing the half-life of glycosylated proteins in *P. pastoris*. This study supports the strategy that the production of aglycosylated forms of some therapeutic Fc-fusion proteins in *P. pastoris* is a feasible approach when facing high cost in mammalian cells. Finally, this study also suggests that IFN-α/Fc-MD or IFN-α/Fc-SC could be a reasonable form of IFN-α/Fc fusion protein expressed in *P. pastoris* for use in further research and applications.

## Additional file

**Additional file 1: Table S1.** Primer sequences used in cloning.

## Abbreviations

IFN: interferon
PEG-IFN-α: pegylated-IFN-α
*P. pastoris*: *Pichia pastoris*
HBV and HCV: hepatitis B and C virus
OAS: oligoadenylate synthetase
FBS: fetal bovine serum
PAS: periodic acid-Schiff
LC–MS: liquid chromatography mass spectrometry
SD: Sprague–Dawley
CBA: cytometric bead array
AOX 1: alcohol oxidase 1
