# Supplementary material for: Elimination of N-glycosylation by site mutation further prolongs the half-life of IFN-α/Fc fusion proteins expressed in Pichia pastoris
Source: Microb Cell Fact. 2016 Dec 7;15:209. doi: 10.1186/s12934-016-0601-9 (PMC5142404; doi:10.1186/s12934-016-0601-9)
Supplement: Supplementary file 1 — Additional file 1: Table S1. Primer sequences used in cloning. [file 12934_2016_601_MOESM1_ESM.doc]

**Table S1. Primer sequences** used in cloning

| **No.** | **Name** | **Sequence** |
| --- | --- | --- |
| 1 | α-Fw | ATGGATCCAAACGATGAGATTTC |
| 2 | Fc-Rv | CTGAATTCTCATTTACCCGGAGACAGGGAGAGG |
| 3 | Sc-Rv | GGAACCACCACCGGAACCACCACCGGAACCTTCCTTACT  TCTTAAACTTTC |
| 4 | Sc-Fw | GGTTCCGGTGGTGGTTCCGGTGGTGGTTCCGCACCTGAA  CTCCTGGGGG |
| 5 | M-Rv | CACGGTACGTGCTTTGGTACTGCTCCTCC |
| 6 | M-Fw | GGAGGAGCAGTACCAAAGCACGTACCGTG |
| 7 | MD-Rv | GGCACGGTGGGCATGTGTGACCACCACCGGAACCACCA  CCACCTTCCTTACTTC |
| 8 | MD-Fw | GTGGTGGTCACACATGCCCACCGTGCCCAGCACCTGAAC  TCCTGGGGGGACCGTCAG |
